# Celiac disease microarray analysis based on System Biology Approach 

**Published:** 2018

**Authors:** Mostafa Rezaei Tavirani, Davood Bashash, Fatemeh Tajik Rostami, Sina Rezaei Tavirani, Abdolrahim Nikzamir, Majid Rezaei Tavirani, Mohammad Hossain Haidary

**Affiliations:** 1 *Proteomics Research Center, Faculty of Paramedical Sciences, Shahid Beheshti University of Medical Sciences, Tehran, Iran*; 2 *Faculty of Medicine, Iran University of Medical Sciences, Tehran, Iran*; 3 *Faculty of Medicine, Shahid Beheshti University of Medical Sciences, Tehran, Iran *

**Keywords:** Celiac disease, System biology, Crucial genes, Cytoscape, ClueGO.

## Abstract

**Aim:**

Aim of this study is screen of the large numbers of related genes of CD to find the key ones.

**Background:**

Celiac disease (CD) is known as a gluten sensitive and immune system dependent disease. There are several high throughput investigations about CD but it is necessary to clarify new molecular aspects mechanism of celiac.

**Methods:**

Whole-genome profile (RNA) of the human peripheral blood mononuclear cells (PBMCs) as Gene expression profile GSE113469 was retrieved Gene Expression Omnibus (GEO) database. The significant genes were selected and analyzed via protein-protein interaction (PPI) network by Cytoscape software. The key genes were introduced and enriched via ClueGO to find the related biochemical pathways.

**Results:**

Among 250 significant genes 47 genes with expressed change above 2 fold change (FC) were interacted and the constructed network were analyzed. The network characterized by poor connections so it was promoted by addition 50 related nodes and 18 crucial nodes were introduced. Two clusters of biochemical pathways were identified and discussed.

**Conclusion:**

There is an obvious conflict between microarray finding and the well-known related genes of CD. This problem can be solve by more attention to the interpretation of PPI ntwork analysis results.

## Introduction

 Celiac as an autoimmune disease is characterized by sensitivity and immune reaction response to gluten component of wheat, rye and barley my se (1). There are evidences that both genetically and environmental factors (gluten) are important elements in relationship with celiac disease (CD) (2). Osteoporosis and iron deficiency anemia are two conditions that the patient may experience due to nutrition deficiency (3, 4). Based on report of Ivor D Hill its occurring in general population is 0.5 – 1 percent (5). Initial serological screening and small intestinal biopsy are the two diagnostic method related to celiac (6). Gluten free nutrition is the keystone treatment for celiac patients (2). Since celiac is genetically a multifactorial disease, roles of HLA and non-HLA genes in this disease is confirmed and are discussed in details (7). 

Today the high throughput methods such as proteomics and genomics which can provide huge values of data or information about diseases are attracted attention of scientists in the medical fields (8-11).Genomics and proteomics studies can provide a high resolution molecular feature of celiac disease. Many informative concepts about molecular mechanism of this disease is obtained by the high throughput investigations (12-15). System biology approaches are effected vastly molecular investigations related to the disease. By using PPI network analysis many unknown molecular aspects of complex diseases can be understand (16). The role of Ubiquitin C, Heat shock protein 90kDa alpha (cytosolic and Grp94); class A, B and 1 member, Heat shock 70kDa protein, and protein 5 (glucose-regulated protein, 78kDa), T-complex, Chaperon in containing TCP1; subunit 7 (beta) and subunit 4 (delta) and subunit 2 (beta) genes in celiac disease is reported via a system biology approach (17). In the network based analysis, the large numbers of elements which are involved in the certain condition are interacted and screened to identify the limited numbers of key elements (18).In this study, the introduced related genes of celiac disease via microarray method will analyze and screen to find possible new molecular aspects of disease and the crucial genes will enrich via gene ontology method. 

## Methods

Gene expression profile GSE113469 was retrieved Gene Expression Omnibus (GEO) database. The profile was provided based on the GPL10558 Illumina HumanHT-12 V4.0 expression bead chip. Whole-genome profile (RNA) of the human peripheral blood mononuclear cells (PBMCs) of celiac patients on gluten free diet (GFD) vs. controls is investigated. The matched patient samples vs. controls were determined via box plot illustration. Numbers of 250 top score genes were selected and differences between control and celiac samples were calculated using the Student’s t test statistical *p*-values less than 0.05 and adjusted *p*-values via GEO2R analysis. Fold change (FC)≥2 was considered to screen the differential expressed genes (DEGs). The uncharacterized DEGs were excluded and the other ones were included to construct a PPI network by using STRING database as a plugin of Cytoscape software version 3.6.0 (19). The network was analyzed and the top10 nodes based on degree value and also betweenness centrality were selected as hub and bottleneck nodes respectively. Interactions between the central nodes is identified by a related sun-network. The central nodes of the celiac network were enriched by KEGG (20) via ClueGO (16). The resulted biochemical pathways were clustered and P-value and also Adjusted P-value less than 0.01were considered. At least presence of 4 genes in term and 2%Gene/Term attribution of nodes in the terms were painstaking. 

**Figure 1:**
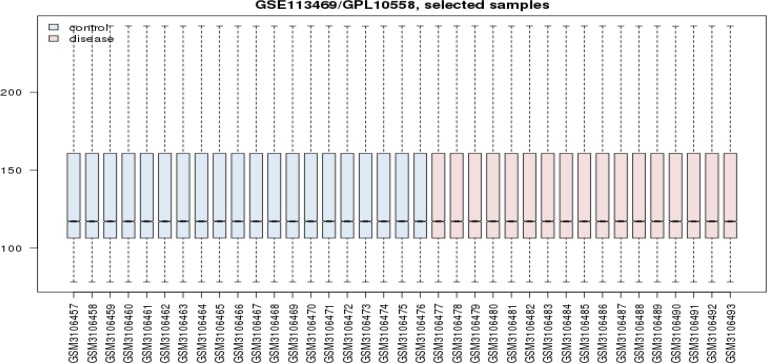
The numbers of 20 control RNA profiles of human PBMCs (blue colored bars) vs. 17 PBMCs of celiac patients on gluten free diet (pink colored bars) are matched via box plot illustration

**Figure 2:**
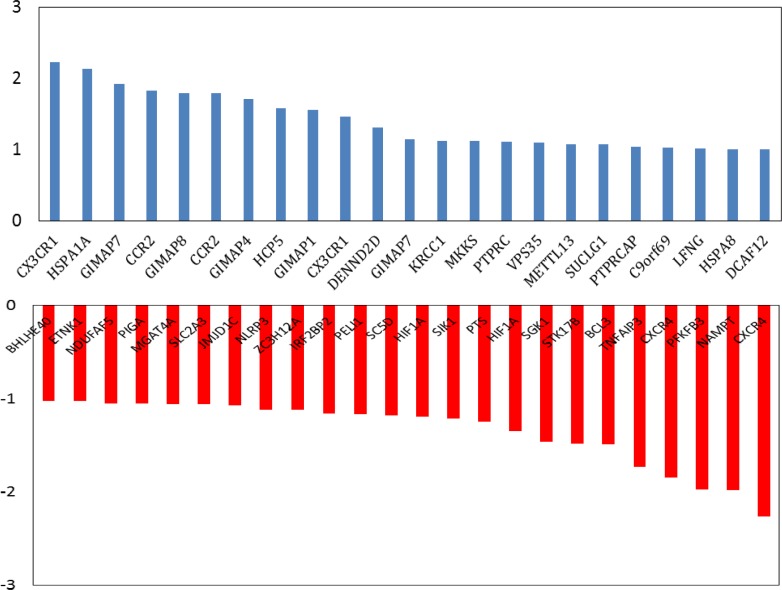
The numbers of 23 up-regulated (blue color) and 24 down-regulated (red color) DEGs of celiac samples vs. controls based on Student’s t test statistical p-values less than 0.05 and adjusted p-values considering FC≥2 were identified. The vertical axis is corresponded to logFC based on 2. The Gene expression was differentially between the GFP patients and control samples

**Figure 3:**
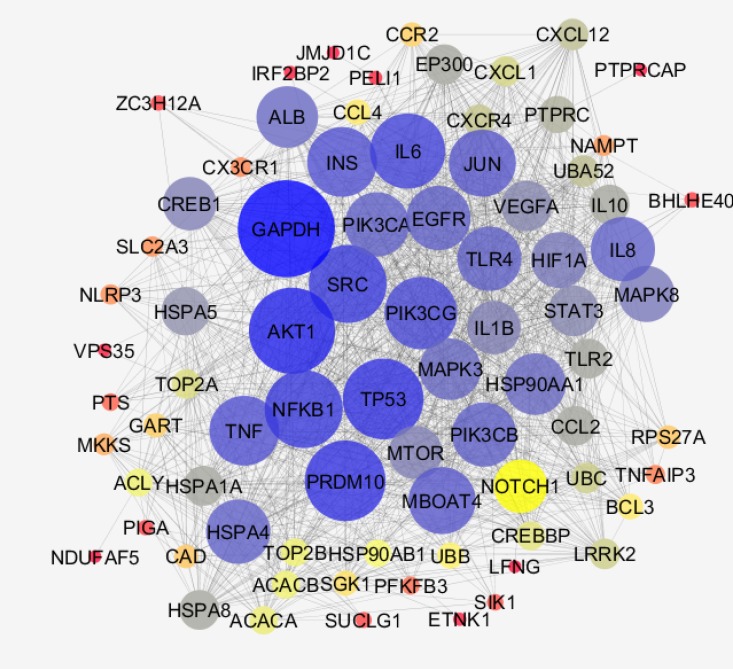
The 41 DEGs and added 50 relate genes characterized by poor connections (the nodes were linked by only 24 edges). After addition 50 related genes (the genes were extracted from STRING database), the network including a main connected component, a component counting 4 nodes, and 8 isolated nodes was designed. The main connected component including 79 nodes and 1243 edges is illustrated

**Table 1:** Rows 1-10 are the hub-nodes and The 1, 3, and 11-18 rows are bottleneck genes of celiac network. The rend color refers to hub-bottleneck nodes and green color is corresponded to bottleneck genes. The query genes are presented as yellow highlighted nodes. The normalized betweenness centrality (NBC) is shown in the last column of table.

**R**	**Gene name**	**description**	**Degree**	**N BC**
1	GAPDH	glyceraldehyde-3-phosphate dehydrogenase	60	0.625
2	AKT1	v-akt murine thymoma viral oncogene homolog 1	56	0.375
3	TP53	tumor protein p53	54	0.813
4	PRDM10	PR domain containing 10	54	0.313
5	SRC	v-src sarcoma (Schmidt-Ruppin A-2) viral oncogene homolog (avian)	53	0.188
6	NFKB1	nuclear factor of kappa light polypeptide gene enhancer in B-cells 1	53	0.156
7	IL6	interleukin 6 (interferon, beta 2)	52	0.281
8	PIK3CG	phosphatidylinositol-4,5-bisphosphate 3-kinase, catalytic subunit gamma	51	0.063
9	INS	Insulin	50	0.313
10	TNF	tumor necrosis factor	50	0.000
11	IL1B	interleukin 1, beta	44	1.000
12	NOTCH1	notch 1	44	0.719
13	HSPA5	heat shock 70kDa protein 5 (glucose-regulated protein, 78kDa)	42	0.563
14	PTPRC	protein tyrosine phosphatase, receptor type, C	38	0.594
15	UBC	ubiquitin C	34	0.469
16	TOP2A	topoisomerase (DNA) II alpha 170kDa	32	0.531
17	ACLY	ATP citrate lyase	29	0.844
18	PELI1	pellino E3 ubiquitin protein ligase 1	3	0.500

**Table 2:** The enriched pathways from KEGG related to the 18 central nodes of celiac disease network are shown. The 22 terms are grouped in 2 clusters (blue and green color terms) which the names of groups are highlighted with yellow color. At least presence of 4 genes in a term and 2%genes/term were considered for term determination. P-value for all identified terms was less than 0.01. The repeated termsare marked by (-1).

R	Term	%Genes/Term	No. of Genes
1	Sphingolipid signaling pathway	3.4	4
2	Apoptosis	2.9	4
3	Longevity regulating pathway	4.5	4
4	Cellular senescence	2.5	4
5	Prolactin signaling pathway	5.7	4
6	Hepatitis C	3.1	4
7	Measles	3.0	4
8	Prostate cancer	4.2	4
9	HIF-1signaling pathway	5.0	5
10	Sphingolipid signaling pathway-1	3.4	4
11	Apoptosis-1	2.9	4
12	Longevity regulating pathway-1	4.5	4
13	Cellular senescence-1	2.5	4
14	Toll-like receptor signaling pathway	3.8	4
15	TNF signaling pathway	3.6	4
16	Insulin resistance	4.7	5
17	Non-alcoholic fatty liver diseases (NAFLD)	3.3	5
18	AGE-RAGE signaling pathway in diabetic complications	4.1	4
19	Chagas disease (American trypanosomiasis	3.8	4
20	Toxoplasmosis	3.5	4
21	Tuberculosis	2.7	5
22	Hepatitis C-1	3.1	4
23	Hepatitis B	4.2	6
24	Measles-1	3.0	4
25	Influenza A	2.3	4
26	Kaposis sarcoma-associated herpesvirus infection	3.3	6
27	Herpes simplex infection	2.1	4
28	Prostate cancer-1	4.2	4
29	Fluid shear stress and atherosclerosis	3.6	5

**Figure 4:**
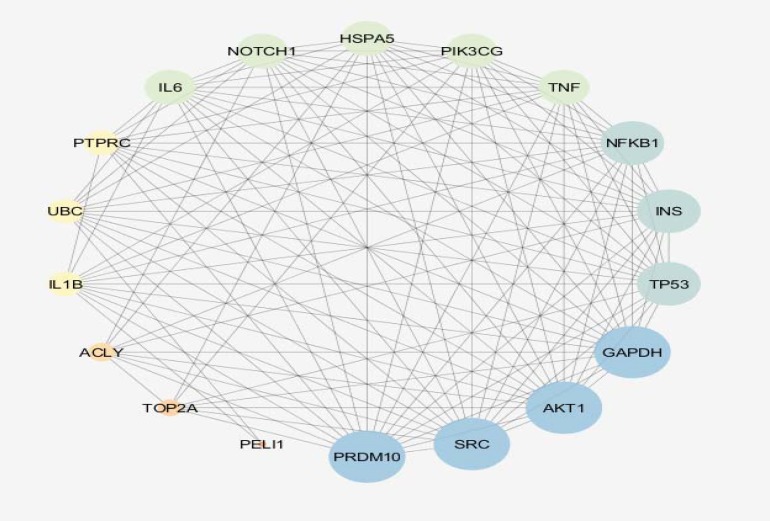
The 18 central nodes of the celiac network are organized in a sub-network. Network is characterized by 117 edges and density equal to 0.765. The nodes are layout by degree value and color from blue to orange corresponds to decrease of degree.

**Figure 5.**
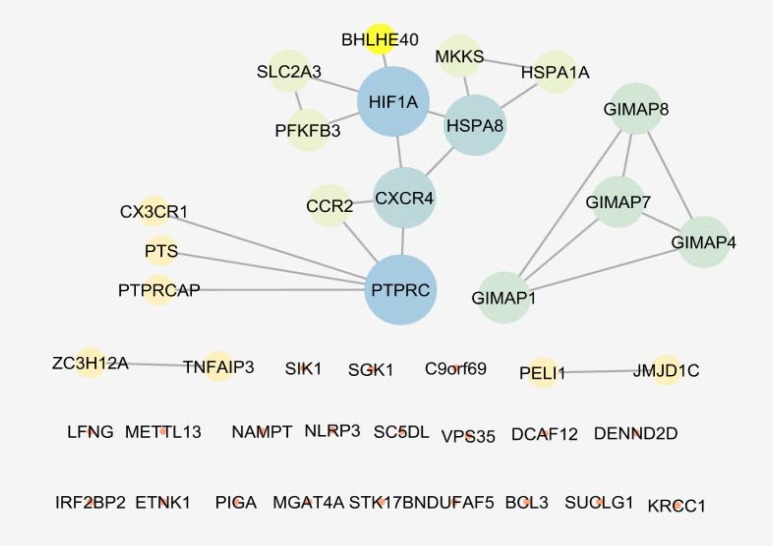
Numbers of 47 DEGs related to celiac disease are interacted. Six genes were not recognized by STRING database and 20 isolated nodes were determined. Two double components and one tetrad were identified. The main connected component included 13 nodes and 16 edges. The nodes are layout by degree value (The bigger size refers to higher degree value.

## Results

As it is shown in the figure 1, 20 control samples are matched with the 17 celiac samples. The midpoints are aligned and samples are comparable. Among 250 top score genes 47 up and down-regulated genes based on statistic method (as described in methods) and considering FC≥2 were identified as the significant DEGs (see figure 2). Therefor 47 DEGs differentiate the GFD patients from control samples. Since 6 DEGs were unknown for STRING database, the numbers of 41 ones were candidate to construct PPI network. The network including the 41 DEGs characterized by poor connections (the nodes were linked by only 24 edges). After addition 50 related genes (the genes were extracted from STRING database (21)), the network including a main connected component, a component counting 4 nodes, and 8 isolated nodes was designed. The main connected component including 79 nodes and 1243 edges is illustrated in the figure 3. The hub and bottleneck nodes are determined and tabulated in the table 1. The 18 central nodes of the network are interacted ant the resulted interacted unit is shown in the figure 4. Density of this sub-network is 0.765 that in compare with density of the main connected component (365) is a higher score and refers to the compact interactions between the central nodes. The enriched pathways from KEGG related to the 18 central nodes of celiac disease network are shown. Number of 22 terms related to the 18 central nodes are identified and clustered (see table 2). At least presence of 4 genes in a term, 2%genes/term, and P-value less than 0.01 were considered. AS it is shown in table 1 only 2 nodes among 18 central nodes are query genes. Therefor the network of merely query genes were analyzed (see figure 5).

## Discussion

Large numbers of data result by high throughput methods in genomics and proteomics which implies to apply suitable screening tools (22). In this research the reported data related to CD were screened by PPI network analysis to find the key elements among them. As it is shown in the figure 1, the samples including CD and control DGEs are statistically comparable. 

By considering restricted condition 47 significant genes were selected for more analysis. 

In the first step it was appear that numbers of up and down-regulated genes are equal approximately and maximum FC is about 5 (see figure 2). CX3CR1 and CXCR4 are remarked as high up and down regulate genes respectively CX3CR1 is receptor of CX3L1/fractalkine which is known as a regulation element of inflammatory response. Relationship between CX3CR1 mutation and crohn,s disease is reported and discussed in details (23). Significant over-expression of this gene also is highlighted in patients on GFD relative to the healthy controls (24). CXCR4 is the other chemo-receptor that its down-regulation is investigated in the several diseases (25-27). Since PPI network analysis showed the there is no considerable connections between the query DEGs, after adding 50 related genes the network was appeared as a scale free network (see figure 3). Network analysis led to introduce 18 central nodes. In the first glance as it is shown in the table 1 it is obvious that except PTPRC and PELI1the other query DEGs were not included among the central genes. However the both mentioned DEGs are not hub-nodes or potent bottleneck genes. The introduced central nodes are connected to each other and constructed a dense sub-network (density is 0.765) (28). The role of hub-genes in the density of this sub-network is prominent. As it is tabulated in the table 1 there are only two hub-bottleneck nodes including GAPDH and TP53 genes. Most of the identified central genes (specially the top hub-nodes) are well-known ones that are involved in different types of cancers, inflammation, and hepatogastro-intestinal diseases (29, 30). The role and correlation between NFKB1 and IL6 genes and CD is investigated and confirm (31, 32). The important point is about several important metabolic related genes such as glyceraldehyde-3-phosphate dehydrogenase, Insulin, and phosphatidylinositol-4, 5-bisphosphate 3-kinase, catalytic subunit gamma as potent central nodes which can effect metabolic features of patients. There are many published research that are concerted by metabolic spected of CD patients (33-35). PELI1 the other DEG that highlighted as central node is known as critical factot for maintenance of peripheral T-cell tolerance. It plays important role in hyper-activation of T-cells (36). 

Protein tyrosine phosphatase, receptor type, C (PTPRC) or (CD45) which is well-known as a regulator of B- and T-cell receptor signaling is one of the DEGs that included in the central nodes list of celiac network (37, 38). 

Gene ontology can provide useful information about roles of a gene set (18, 39). The enriched biochemical pathways related to the central nodes of celiac network (table 2) indicate that two clusters of pathways are involved in CD. Prolactin signaling pathway including Sphingolipid signaling pathway, Apoptosis, Longevity regulating pathway, Cellular senescence, Prolactin signaling pathway, Hepatitis C, Measles, and Prostate cancer is the first cluster. Number of 21 pathways (including 7 common pathways with cluster-1) are related to cluster-2. Therefor except Prolactin signaling pathway all pathways of first cluster are common with cluster-2. Eight pathways are related directly to response to viruses. It is obvious that viruses activate immune and inflammatory systems in body (40-42). Cellular Senescence; the extremely cell cycle arrest which protect cell vs. cancer progression characterized by barrier formation against proliferation of damaged cell (43) and apoptosis are the two other pathways that are determined. Hypoxia-inducible factor-1is a mediator that is involved in the response to the reduced O2 condition (44). Presence of several metabolic and inflammatory pathways among the identified pathways correspond to the characteristic property of CD. 

As it is mentioned in the result part the network including the 47 query DEGs was a poor network by considering connections between the nodes even the numbers of six genes were not recognized by STRING database. Again the network was analysis (see figure 5) and its details were studied. The network includes 20 isolated nodes (the nodes without any connection), two double components (four nodes and two connection), one tetrad (four nodes and 6 edges), and a main connected component included 13 nodes and 16 edges. There is a conflict of presence as central nodes between the query DEGs and the additional related genes. This point may be resulted from more information about binding properties of the related genes relative to the query DEGs. The seven top central nodes which are “related gens” were searched by Google search engine by key words including name of genes as like “GAPDH gene”. The obtained documents for GAPDH, AKT1, TP53, PRDM10, SRC, NFKB1, and IL6 were as 56,800,000, 273,000, 1,160,000, 30,700, 50,800,000, 63900, and 58,600,000 respectively. In the similar search for the seven top up-regulated genes; CX3CR1, HSPA1A, GIMAP7, CCR2, GIMAP8, GIMAP4, and HCP5 the numbers of documents were as: 158,000, 36,000, 23,300, 211,000, 29500, 36100, and 29600 respectively. It can be concluded that more information and also details of properties may effect on the arrangement of the nodes of the network. Therefor in addition to the central nodes the significant DEGs should be considered to obtain a more precious description of disease. 

In addition to introduce a possible biomarker panel for celiac disease, it was suggested that the analyzed and screened significant Differential expressed genes should be considerd as important players in the pathology of celiac disease.
